# Supplementary material for: Language-Related Disparities in Pain Management in the Post-Anesthesia Care Unit for Children Undergoing Laparoscopic Appendectomy
Source: Children (Basel). 2020 Oct 4;7(10):163. doi: 10.3390/children7100163 (PMC7600632; doi:10.3390/children7100163)
Supplement: Supplementary file 1 [file children-07-00163-s001.pdf]

children

MDPI

Supplementary

Table S1. Intraoperative opioid administration (in OME per kilogram), by English proficiency.

	**Adjusted Estimate (95%CI)***
Low English proficient (ref = English proficient)	1.07 (0.70,1.59)
Age in years	0.99 (0.95,1.03)
Gender (ref = male)	0.96 (0.70,1.32)

Abbreviations: OME, oral morphine equivalents; CI, confidence interval. * Coefficients were also adjusted for race (White, Black, Asian, and other) but these are not reported given the small cohort size of some racial subgroups.
